# Supplementary material for: The 2.3 Å Structure of A21, a Protein Component of the Conserved Poxvirus Entry-Fusion Complex
Source: bioRxiv. 2025 Jan 8:2025.01.08.631918. Preprint. [Version 1] doi: 10.1101/2025.01.08.631918 (PMC12190314; doi:10.1101/2025.01.08.631918)
Supplement: Supplement 2 [file NIHPP2025.01.08.631918v1-supplement-2.pdf]

bioRxiv preprint doi: https://doi.org/10.1101/2025.01.08.631918; this version posted January 9, 2025. The copyright holder for this preprint (which was not certified by peer review) is the author/funder. This article is a US Government work. It is not subject to copyright under 17 USC 105 and is also made available for use under a CC0 license.

Table S1. A21 data collection and refinement statistics

	**PDB ID: 8U0R**
**Data collection**	
Space group	$P2_1$
Cell dimensions	
a, *b*, *c* (Å)	46.86, 70.06, 78.95
α, β, γ (°)	90.00, 95.29, 90.00
Resolution (Å)	39.31 - 2.30 (2.38 - 2.30)*
R_{sym} or R_{merge}	0.101 (0.333)
$I / \sigma I$	8.2 (3.1)
Completeness (%)	99.2 (92.2)
Redundancy	3.72 (3.25)
**Refinement**	
Resolution (Å)	39.31 - 2.30
No. reflections	22,602
R_{work} / R_{free}	0.211/0.239
No. atoms	
Protein	3,389
Ligand/ion	213
Water	85
B-factors [Å^2]	
Protein	50.69
Ligand/ion	56.29
Water	50.16
R.m.s deviations	
Bond lengths (Å)	0.011
Bond angles (°)	1.301

*Highest resolution shell is shown in parenthesis.

bioRxiv preprint doi: https://doi.org/10.1101/2025.01.08.631918; this version posted January 9, 2025. The copyright holder for this preprint (which was not certified by peer review) is the author/funder. This article is a US Government work. It is not subject to copyright under 17 USC 105 and is also made available for use under a CC0 license.

Table S2. Hydrogen bonds occurring in A21 loops

A21 atoms	Hydrogen bond length (Å)
Loop 1	
**Asn54 (N)···Lys61 (O)***	3.0
**Asn54 (O)···Lys61 (N)**	3.0
Asn54 (OD1)···Lys63 (NZ)	2.5
Asn54 (OD1)···Tyr91 (OH)	3.0
Loop 2	
**Ala65 (O)···Ile73 (N)**	3.2
**Asp67 (N)···Val71 (O)**	2.7
**Asp67 (O)···Asn70 (N)**	2.8
Asp67 (OD1)···**Lys69 (N)**	2.9
Asp67 (OD1)···**Val71 (N)**	2.7
**Pro74 (O)···Lys77 (N)**	3.2
Loop 3	
**Asp93(O)···Arg96 (N)**	3.1
**Ile99 (O)···Phe102 (N)**	3.3
**Gly101 (O)···Ser105 (N)**	3.0
**Ala62 (O)···Cys92 (N)**	2.8
Asp93 (OD1)···**Asp95 (N)**	2.6
Arg96 (NH1)···Asp98 (OD1)	3.0
Arg49 (NH1)···**Pro100 (O)**	3.3
Arg49 (NH2)···**Pro100 (O)**	2.8
Ser105 (OG)···**Gly101 (O)**	3.0

*Main chain N and O hydrogen bonds are given in bold.
